# Simulation of the Peritectic Phase Transition in Fe-C Alloys

**DOI:** 10.3390/ma15020537

**Published:** 2022-01-11

**Authors:** Hui Fang, Qianyu Tang, Qingyu Zhang, Yiming Fan, Shiyan Pan, Markus Rettenmayr, Mingfang Zhu

**Affiliations:** 1 Jiangsu Key Laboratory of Advanced Metallic Materials, School of Materials Science and Engineering, Southeast University, Nanjing 211189, China; hui.fang@seu.edu.cn (H.F.); qianyu_soup@126.com (Q.T.); fym19960928@163.com (Y.F.); 2 Shagang School of Iron and Steel, Soochow University, Suzhou 215137, China; qingyu.zhang@suda.edu.cn; 3 School of Materials Science and Engineering, Nanjing University of Science and Technology, Nanjing 210094, China; calculate@163.com; 4 Otto Schott Institute of Materials Research, Friedrich-Schiller-Universität, Löbdergraben 32, 07743 Jena, Germany; m.rettenmayr@uni-jena.de

**Keywords:** peritectic solidification, Fe-C alloys, diffusion, phase transformation kinetics, multi-phase cellular automaton

## Abstract

In this work, a multi-phase cellular automaton (CA) model is extended for the quantitative simulation of peritectic phase transition. First, the effects of cooling rate/supersaturation and temperature on the peritectic transformation kinetics in Fe-C alloys are investigated by utilizing the present CA model. The CA simulations show that supersaturations in the parent phases (liquid and δ-ferrite) increase the L/γ interface growth velocity remarkably, but tinily for the δ/γ interface migration velocity. There exists a transition supersaturation for isothermal transformations, at which the growth rates of the two interfaces are equal. The transition supersaturation is found to increase with decreasing temperature. Microstructural evolution at different cooling rates during peritectic transformation is simulated using the experimental conditions. At low cooling rates, the δ/γ interface propagates at a higher velocity than the L/γ interface. At high cooling rates, however, the γ-phase grows more into the L-phase with a cellular morphology. Then, the proposed CA model is applied to simulate the microstructural evolution during peritectic reaction. It is observed that the γ-phase propagates along the L/δ interface and finally encircles the δ-phase. Meanwhile, the intervenient γ-phase grows in thickness through peritectic transformation. The CA simulations are compared reasonably well with the experimental data and analytical calculations.

## 1. Introduction

Peritectic phase transition, involving the peritectic reaction (L + δ→γ, namely the γ-phase growth along the L/δ interface) and the peritectic transformation (L→γ and δ→γ, namely the γ-phase thickening into the L- and δ-phases) [1], has attracted great interest in both academic study and industrial production. The γ-phase growth kinetics significantly influences the peritectic microstructures and, thereby, the mechanical properties of the final products [2,3].

Extensive experiments have been carried out to study the peritectic solidification of Fe-C alloys by utilizing the solid/liquid diffusion couple method [4,5,6] and high temperature laser scanning confocal microscopy (HTLSCM) combined with the concentric solidification technique [3,7,8,9]. Griesser et al. [9] studied peritectic solidification of Fe-C and Fe-Ni alloys under the conditions close to thermal and chemical equilibrium. It is confirmed that both the peritectic reaction and peritectic transformation are governed by a diffusion-controlled mechanism. The thicknesses of the γ-phase formed during isothermal peritectic transformation under different temperatures were measured in experiments by using the solid/liquid diffusion couple method [4,5,6]. It is reported that the migration of both the L/γ and δ/γ interfaces obeys the parabolic law. Moreover, the fraction of the γ-phase solidified from the liquid phase is less than that transformed from the δ-phase under isothermal condition. The peritectic transformation in Fe-C alloys under a continuous cooling condition has also been studied experimentally. Phelan et al. [8] performed in situ observation experiments employing HTLSCM to figure out the influence of the cooling rate on the peritectic transformation kinetics. It was found that at the low cooling rate of ~10 K/min, the δ/γ interface migrated faster than the L/γ interface, while the L/γ interface propagated at a higher velocity when the cooling rate reached ~100 K/min. Yet, the mechanism of the experimentally observed phenomena remained unclear.

Theoretical studies have also been performed for the peritectic transformation [10,11] and reaction [12]. Some of the present authors have recently proposed an analytical model that considers solute diffusion in the δ-, γ- and L-phases, which is able to explicitly solve the time-dependent γ-phase thickness and solute concentration distributions in the δ-, γ- and L-phases [11]. The predicted parabolic rate constants agree well with experimental results. However, the application of analytical model is limited to one-dimensional (1D) peritectic transformation under isothermal condition.

Numerical modeling, as a powerful tool to analyze the solidification process at a detailed level, has been widely adopted to describe complex phase transformation phenomena in recent years. Various phase-field (PF) simulations were carried out to study the peritectic phase transition of Fe-C alloys [8,11,13,14,15,16,17]. Ohno and Matsuura [15] performed 1D PF simulations to simulate the isothermal peritectic transformation at different holding temperatures. Pan and Zhu [11] studied the isothermal γ-phase growth kinetics during peritectic transformation at different temperatures and supersaturations. Phelan et al. [8] performed 1D PF simulations at different cooling rates. It was suggested that cooling rates influence the solute buildup at the interfaces and, hence, the propagation velocities at the L/γ and δ/γ interfaces. However, quantitative PF calculation requires huge computation resources and, thus, are concentrated on 1D isothermal peritectic transformation with zero or small supersaturations. Moreover, the system size and transformation time utilized in the quantitative PF studies (e.g., 7 μm and 10 ms in Ohno and Matsuura’s work [15]; 100 μm and 2 s in Pan and Zhu’s work [11]) are of several magnitudes smaller than the spatial and temporal scales in experiments (e.g., 7–15 mm and 15–29 ks [4,5]). The influence of cooling rates on the morphology of γ-phase still needs further verification.

Cellular automaton (CA) models are capable of simulating lots of experimentally observed solidification microstructures [18,19,20,21,22,23,24,25,26]. The CA method has also been applied to simulate peritectic microstructures [27,28,29,30]. Su et al. [27] and Yamazaki et al. [28] simulated the microstructural evolution of a C-Mn steel and Fe-C alloy during peritectic solidification. The fraction of the γ-phase during peritectic transformation was calculated by the Scheil model [27] and lever rule [28] in accordance with the temperature, respectively. Ogawa and Natsume [29] performed the CA simulations regarding the microstructural evolution of hypo- and hyper-peritectic Fe-C alloys at a cooling rate of 10 K/s. We recently proposed a quantitative multi-phase CA model that can simulate the microstructural evolution during peritectic transformation [30]. The isothermal growth kinetics of the γ-phase is studied at the experimental time (30 ks) and length (12 mm) scales. The validity of the proposed quantitative CA model is confirmed by comparing with the analytical solution and experimental data. Nevertheless, in all the above CA and PF simulations, the influences of the cooling conditions on the transition of the γ-phase growth rate into the two parent phases and the morphology of the γ-phase have so far not been investigated.

In the present work, the multi-phase CA model recently proposed by the present authors [30] is extended for studying the peritectic phase transition, including both peritectic transformation and peritectic reaction. The effects of temperature and cooling condition on the peritectic γ-phase growth kinetics and morphology are verified by CA simulation. The inversion of the L/γ and δ/γ interface growth velocities and the transition holding temperature/supersaturation are quantitatively analyzed. Microstructural evolution at different cooling rates during peritectic transformation is simulated using the experimental conditions. The influence of cooling rates on the γ-phase morphology is studied. Microstructural evolution during peritectic reaction under the experimental conditions is also simulated. The simulation results are compared with the analytical predictions and experimental data.

## 2. Governing Equations of the Multi-Phase CA Model and Computation Procedure

In this section, the governing equations of the extended multi-phase CA model are provided, and the computation procedure for the simulation of both peritectic transformation and peritectic reaction is described in detail.

### 2.1. Governing Equations

The simulation domain is divided into uniform grids. In a peritectic solidification system, the state of a grid includes the γ-phase (*f*_γ_ = 1), L-phase (*f*_L_ = 1), δ-phase (*f*_δ_ = 1), L/δ interface (*f*_L_ + *f*_δ_ = 1), L/γ interface (*f*_L_ + *f*_γ_ = 1), δ/γ interface (*f*_δ_ + *f*_γ_ = 1) and L/γ/δ triple junction (*f*_L_ + *f*_γ_ + *f*_δ_ = 1) that can be regarded as a combination of the L/δ, L/γ and δ/γ interfaces. The density change during peritectic solidification is neglected in the present work. The moving rates of the L/δ, L/γ and δ/γ interfaces are calculated on the basis of a local equilibrium approach [31]. Within a time interval, Δ*t*, the phase fraction increment in an interface grid at each interface is calculated by:

(1) $$L/\delta \;\mathrm{interface}:\;\Delta f_{\delta }^{L/\delta }=G_{L/\delta }\cdot \frac{C_{L,L/\delta }^{\mathrm{eq}}-C_{L,L/\delta }^{*}}{C_{L,L/\delta }^{\mathrm{eq}}(1-k_{L/\delta })},$$

(2) $$L/\gamma \;\mathrm{interface}:\;\Delta f_{\gamma }^{L/\gamma }=G_{L/\gamma }\cdot \frac{C_{L,L/\gamma }^{\mathrm{eq}}-C_{L,L/\gamma }^{*}}{C_{L,L/\gamma }^{\mathrm{eq}}(1-k_{L/\gamma })},$$

(3) $$\delta /\gamma \;\mathrm{interface}:\;\Delta f_{\gamma }^{\delta /\gamma }=G_{\delta /\gamma }\cdot \frac{C_{\delta ,\delta /\gamma }^{\mathrm{eq}}-C_{\delta ,\delta /\gamma }^{*}}{C_{\delta ,\delta /\gamma }^{\mathrm{eq}}(1-k_{\delta /\gamma })},$$

where $C_{L,L/\delta }^{\mathrm{eq}}$, $C_{L,L/\gamma }^{\mathrm{eq}}$ and $C_{\delta ,\delta /\gamma }^{\mathrm{eq}}$ are the equilibrium concentrations, and $C_{L,L/\delta }^{*}$, $C_{L,L/\gamma }^{*}$ and $C_{\delta ,\delta /\gamma }^{*}$ are the local actual concentrations at the L/δ, L/γ and δ/γ interfaces, respectively; *k*_L/δ_, *k*_L/γ_ and *k*_δ/γ_ are the solute partition coefficients, and *G*_L/δ_, *G*_L/γ_ and *G*_δ/γ_ are the geometrical factors at the L/δ, L/γ and δ/γ interfaces, respectively. The geometrical factors, related to the states of neighboring grids, are incorporated in the 2D simulations for eliminating the CA square mesh-induced artificial anisotropy [30,32]. The equations for calculating *G*_L/δ_ are given below as an example:

(4) $$G_{L/\delta }=\min\left(\left(\sum_{m=1}^{4}S_{m}^{I}+\frac{1}{\sqrt{2}}\sum_{m=1}^{4}S_{m}^{II}\right)/6,1/3\right),$$

$$\mathrm{solidification}:\;S^{I},S^{II}=\left\{ \begin{array}{l} 0\;\;\;(f_{\delta }<1)\\ 1\;\;\;(f_{\delta }=1) \end{array}\right.,$$

$$\mathrm{melting}:\;S^{I},S^{II}=\left\{ \begin{array}{l} 0\;\;\;(f_{\delta }>0)\\ 1\;\;\;(f_{\delta }=0) \end{array}\right.,$$

where *m* represents the four nearest and four second-nearest grids in the Moore-type neighborhood, *S^I^* and *S^II^* represent the states of the nearest and second-nearest neighboring grids and *f*_δ_ is the fraction of δ-phase. Substituting *f*_δ_ in Equation (4) to *f*_γ_ gives the calculation equation for *G*_L/γ_ and *G*_δ/γ_, respectively.

The local equilibrium concentration at the interfaces are given by:

(5) $$L/\delta \;\mathrm{interface}:\;C_{L,L/\delta }^{\mathrm{eq}}=\frac{T^{*}-T_{m,L/\delta }}{m_{L/\delta }}+\frac{\Gamma_{L/\delta }K_{L/\delta }}{m_{L/\delta }},$$

(6) $$L/\gamma \;\mathrm{interface}:\;C_{L,L/\gamma }^{\mathrm{eq}}=\frac{T^{*}-T_{m,L/\gamma }}{m_{L/\gamma }}+\frac{\Gamma_{L/\gamma }K_{L/\gamma }}{m_{L/\gamma }},$$

(7) $$\delta /\gamma \;\mathrm{interface}:\;C_{\delta ,\delta /\gamma }^{\mathrm{eq}}=\frac{\left(T_{p}-T_{m,L/\delta })\cdot (T^{*}-T_{m,\delta /\gamma }\right)}{\left(T_{p}-T_{m,\delta /\gamma })\cdot (m_{L/\delta }/k_{L/\delta }\right)}+\frac{\Gamma_{\delta /\gamma }K_{\delta /\gamma }}{m_{\delta /\gamma }},$$

where T* is the local actual temperature at the interface; *T_p_* is the peritectic temperature; *T_m_*_,L/δ_ and *T_m_*_,L/γ_ are the melting points of the pure bcc-Fe and fcc-Fe, respectively; *T_m_*_,δ/γ_ is the δ/γ transition temperature of pure Fe; *m*_L/δ_ and *m*_L/γ_ are the liquidus slopes of the δ- and γ-phases, respectively; *m*_δ/γ_ is the slope of the δ/γ phase boundary; Γ_L/δ_, Γ_L/γ_ and Γ_δ/γ_ are the Gibbs–Thomson coefficients, and *K*_L/δ_, *K*_L/γ_ and *K*_δ/γ_ are the interfacial curvatures of the L/δ, L/γ and δ/γ interfaces, respectively; the equation for calculating *K*_L/δ_ is given below as an example:

(8) $$K_{L/\delta }={\left[{\left(\frac{\partial f_{\delta }}{\partial x}\right)}^{2}+{\left(\frac{\partial f_{\delta }}{\partial y}\right)}^{2}\right]}^{-\frac{3}{2}}\times \left[2\frac{\partial f_{\delta }}{\partial x}\frac{\partial f_{\delta }}{\partial y}\frac{\partial^{2}f_{\delta }}{\partial x\partial y}-{\left(\frac{\partial f_{\delta }}{\partial x}\right)}^{2}\frac{\partial^{2}f_{\delta }}{\partial y^{2}}-{\left(\frac{\partial f_{\delta }}{\partial y}\right)}^{2}\frac{\partial^{2}f_{\delta }}{\partial x^{2}}\right],$$

Substituting *f*_δ_ in Equation (8) to *f*_γ_ gives the calculation equation for *K*_L__/__γ_ and *K*_δ__/__γ_, respectively.

The local actual liquid concentrations are determined from the solute redistribution and diffusion, calculated by:

(9) $$\frac{\partial C}{\partial t}=\nabla \left(D\left(f_{\gamma },f_{\delta }\right)\nabla (C/p(f_{\gamma },f_{\delta }))\right),$$

where *D*(*f*_γ_,*f*_δ_) is the diffusion coefficient corresponding with phase fractions and calculated by *D*(*f*_γ_,*f*_δ_) = *k*_L__/γ_*D*_γ_*f*_γ_ + *k*_L__/δ_*D*_δ_*f*_δ_ + *D*_L_(1−*f*_γ_−*f*_δ_), where *D*_γ_, *D*_δ_ and *D*_L_ are the diffusion coefficients in the γ-, δ- and L-phases, respectively; *p*(*f*_γ_,*f*_δ_) is a conversion coefficient function calculated by *p*(*f*_γ_,*f*_δ_) = *k*_L__/γ_*f*_γ_ + *k*_L__/δ_*f*_δ_ + (1−*f*_γ_−*f*_δ_). A zero flux boundary condition is adopted at four walls of the two-dimensional simulation domain. The time step is calculated using Δ*t* = Δ*x*^2^/5*D*_L_, where Δ*x* is the mesh size. The thermodynamic and physical parameters of Fe-C alloys used in this study can be found elsewhere [11].

### 2.2. Computation Procedure

As described above, different cooling rates will yield different levels of solute buildup in the parent L- and δ-phases, leading to different growth rates at the L/γ and δ/γ interfaces [8]. The level of solute buildup can be characterized by the supersaturation, defined as $\Omega_{\delta }=(C_{\delta ,\delta /\gamma }^{\mathrm{eq}}-C_{\delta ,\infty }^{})/(C_{\delta ,\delta /\gamma }^{\mathrm{eq}}-C_{\gamma ,\delta /\gamma }^{\mathrm{eq}})$ and $\Omega_{L}=(C_{L,L/\gamma }^{\mathrm{eq}}-C_{L,\infty }^{})/(C_{L,L/\gamma }^{\mathrm{eq}}-C_{\gamma ,L/\gamma }^{\mathrm{eq}})$, where Ω_δ_ and Ω_L_ represent the supersaturations in the δ-phase and L-phase, and *C*_δ,__∞_ and *C*_L,__∞_ are the concentrations of the δ-phase and L-phase at the locations far away from the interface, respectively. At low cooling rates, complete solute diffusion in the parent phases is assumed, which is equivalent to zero supersaturation, Ω_δ_ = Ω_L_ = 0. Evidently, Ω_δ_ and Ω_L_ increase when the cooling rate is increased.

Thus, simulations are first performed with different supersaturations to investigate how the cooling rate influences the peritectic transformation kinetics. The CA simulation results are compared with analytical solutions and experimental data for validation. Then, the experimental cooling conditions are adopted in CA simulations to study the effect of cooling rate on the growth kinetics and γ-phase morphology. Finally, the microstructural evolution during peritectic reaction is studied and compared with in situ experimental observation.

In the simulations of peritectic reaction, at the beginning, the domain contains the δ- and L-phases. A γ-phase seed is assigned to the L/δ interface, then the γ-phase grows along the L/δ interface, which is led by the L/γ/δ triple junction. On the other hand, in the simulations of peritectic transformation, the γ-nucleus is initially set between the δ- and L-phases and the two parent phases are completely separated. Thus, the coexistence of three phases, i.e., the peritectic reaction, is neglected in the simulations of peritectic transformation.

**Figure 1:**
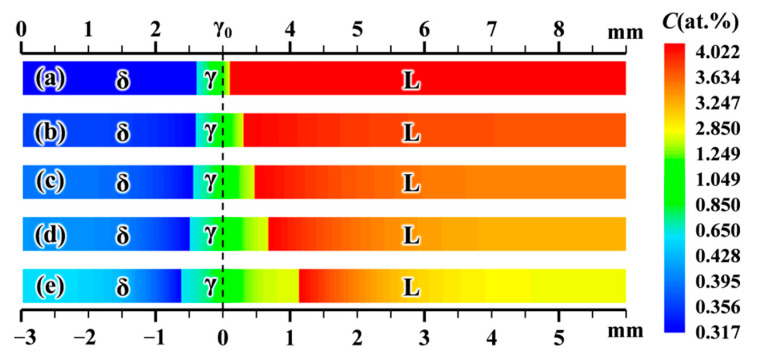
Simulated thicknesses of γ-platelets and carbon concentration distribution after holding for 100 s at 1744 K at different supersaturations: (**a**) Ω = 0, (**b**) Ω = 0.15, (**c**) Ω = 0.25, (**d**) Ω = 0.35, (**e**) Ω = 0.5. The black dashed line indicates the initial γ-nucleus position.

**Figure 2:**
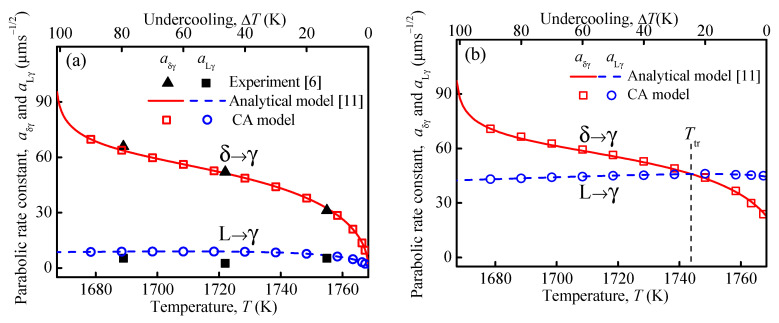
Comparison among CA simulations, analytical predictions [11] and experimental data [6] regarding the parabolic rate constants *a*_Lγ_ and *a*_δγ_ changing with holding temperature under the conditions of: (**a**) Ω = 0, (**b**) Ω = 0.25.

**Figure 3:**
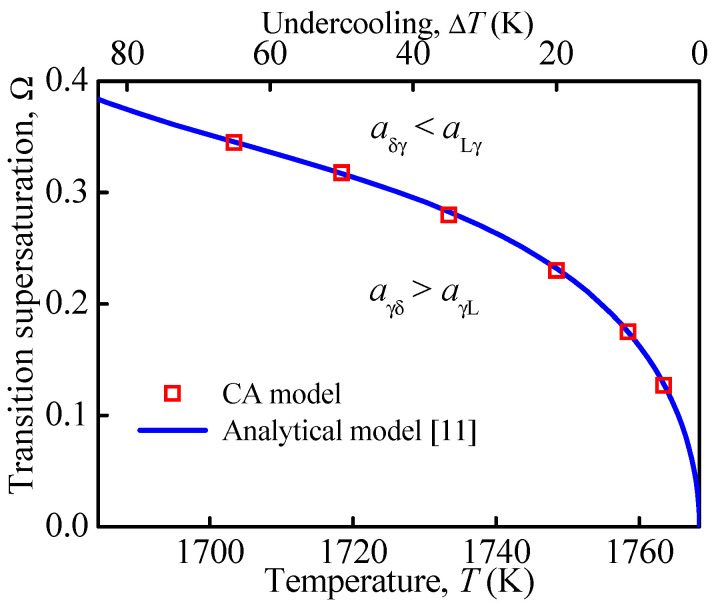
Comparison of transition supersaturation as a function of holding temperature between the CA and analytical models [11].

For simulations of peritectic transformation with different supersaturations (Figure 1, Figure 2, Figure 3), the computation domain is a 9000 × 2 mesh with Δ*x* = 1 μm. Using such a domain size, the domain boundary will not influence the solute diffusion boundary layers ahead of the L/γ and δ/γ interfaces for the five supersaturations of Ω = Ω_δ_ = Ω_L_ = 0, 0.15, 0.25, 0.35 and 0.5 chosen for the simulations. The peritectic transformation is assumed to occur isothermally at a holding temperature of *T*_0_ = 1744 K, which is below the peritectic temperature of *T_p_* = 1768.4 K (Δ*T* = *T_p_* − *T*_0_ = 24.4 K). Initially, the system consists of the δ- and L-phases. A γ-nucleus with an initial width of 4 μm is set between the δ- and L-phases. The equilibrium concentrations calculated by Equations (6) and (7) are $C_{L,L/\gamma }^{\mathrm{eq}}$ = 4.08 at.% at the plane L/γ interface and $C_{\delta ,\delta /\gamma }^{\mathrm{eq}}$ = 0.32 at.% at the plane δ/γ interface. The initial concentrations of the parent phases under different conditions are calculated according to the definition of the supersaturations (e.g., $C_{\delta ,0}=C_{\delta ,\delta /\gamma }^{\mathrm{eq}}$ = 0.32 at.% and $C_{L,0}=C_{L,L/\gamma }^{\mathrm{eq}}$ = 4.08 at.% for Ω = 0; *C*_δ,0_ = 0.45 at.% and *C*_L,0_ = 2.72 at.% for Ω = 0.5). The initial concentration of the γ-nucleus changes from $C_{\gamma ,\delta /\gamma }^{\mathrm{eq}}=k_{\delta /\gamma }C_{\delta ,\delta /\gamma }^{\mathrm{eq}}$ = 0.59 at.% at the δ/γ interface to $C_{\gamma ,L/\gamma }^{\mathrm{eq}}=k_{L/\gamma }C_{L,L/\gamma }^{\mathrm{eq}}$ = 1.36 at.% at the L/γ interface linearly. With the peritectic transformation proceeding, the parent phases are gradually consumed by the growth of the γ-phase. The width of the γ-phase grown into the L- and δ-phases are designated as *d*_Lγ_ and *d*_δγ_, respectively.

It is known that for diffusion-controlled peritectic solidification, using valid diffusivities in the three phases is important for predicting the γ-phase growth kinetics. Ohno & Matsuura [15] and Pan & Zhu [11] carried out quantitative PF simulations to study the isothermal peritectic transformation kinetics of Fe-C alloys, and the simulated parabolic rate constants in their work agree well with the experimental measurements. Thus, in the present work, the temperature-dependent diffusivities used in Refs. [11,15] are adopted in CA simulations.

## 3. Results and Discussion

### 3.1. Growth Kinetics and Microstructural Evolution during Peritectic Transformation

Figure 1 presents the simulated distances that the γ-phase (green to yellow) grows into the parent phases, and carbon concentration distributions in the three phases after holding for 100 s at different supersaturations. The black dashed line represents the initial location of the γ-nucleus. The left-hand side is the δ-phase (blue) and the right-hand side is the L-phase (red to yellow). As shown, for Ω = 0 (Figure 1a), the concentrations are uniform in the parent phases, while they are inhomogeneous in the γ-phase. For Ω > 0 (Figure 1b–e), at the left-hand side (δ-phase), the concentration increases from $C_{\delta ,\delta /\gamma }^{*}$ at the δ/γ interface to *C*_δ,∞_ at the left wall of the domain. Conversely, at the right-hand side (L-phase), the concentration decreases from $C_{L,L/\gamma }^{*}$ at the L/γ interface to *C*_L,∞_ at the right wall. The total width of the γ-phase in the horizontal direction increases with increasing supersaturation, in which *d*_Lγ_ increases significantly and *d*_δγ_ only slightly. In the case of low supersaturations, *d*_δγ_ is larger than *d*_Lγ_. When the supersaturation is increased to Ω ≥ 0.25, the opposite is observed, *d*_Lγ_ is larger than *d*_δγ_. Since the peritectic transformation is diffusion controlled, the γ-phase growth kinetics is directly related to the diffusion flux, $J_{j}=-D_{j}\partial_{x}C_{j}(j=\delta ,\gamma ,L)$, at the two interfaces [11]. It is found that when the supersaturation (i.e., solute buildup in the parent phases) is increased from Ω = 0 to Ω = 0.5, at the L/γ interface, the diffusion flux increasement is about 30 at.%μm/s, while it is only ~1.5 at.%μm/s at the δ/γ interface for the case of *d*_γ_ = 925 μm. Thus, as expected, the impact of supersaturation on the phase transformation kinetics is more pronounced at the L/γ interface than at the γ/δ interface since the solute diffusivity in the L-phase is larger than that in the δ-phase.

In isothermal peritectic transformation experiments, the parabolic rate constants, defined by *a*_Lγ_ = $d_{L\gamma }/\sqrt{t}$ and *a*_δγ_ = $d_{\delta \gamma }/\sqrt{t}$, are usually used to characterize the growth kinetics of the γ-phase [6]. CA simulations were performed at different isothermal holding temperatures for the two supersaturations of Ω = 0 and Ω = 0.25, see Figure 2. Other conditions used in the simulation were the same as those in Figure 1. In Figure 2, the parabolic rate constants, *a*_δγ_ and *a*_Lγ_, varying with the holding temperature, *T*_0_, are presented as obtained from the CA model, analytical model [11], and the experimental data (Ω = 0) [6]. As shown, the simulated results and the analytical predictions are nearly identical for both *a*_δγ_ and *a*_Lγ_ for both Ω = 0 and Ω = 0.25. The CA profile with Ω = 0 also compares well with the experimental results in Figure 2a. The good agreement demonstrates the capability of quantitative predictions of the present multi-phase CA model.

It is noted from Figure 2 that *a*_δγ_ increases remarkably with decreasing holding temperature, while *a*_Lγ_ remains nearly unchanged for both Ω = 0 and Ω = 0.25. For Ω = 0, the growth rates at the δ/γ and L/γ interfaces are proportional to $\partial_{x}C_{\gamma }{|}_{{\gamma \delta }^{+}}/(C_{\gamma ,\delta /\gamma }^{\mathrm{eq}}-C_{\delta ,\delta /\gamma }^{\mathrm{eq}})$ and $\partial_{x}C_{\gamma }{|}_{{\gamma L}^{-}}/(C_{L,L/\gamma }^{\mathrm{eq}}-C_{\gamma ,L/\gamma }^{\mathrm{eq}})$, respectively [11], where $\partial_{x}C_{\gamma }$ represents the concentration gradient, and the denominators, $C_{\gamma ,\delta /\gamma }^{\mathrm{eq}}-C_{\delta ,\delta /\gamma }^{\mathrm{eq}}$ and $C_{L,L/\gamma }^{\mathrm{eq}}-C_{\gamma ,L/\gamma }^{\mathrm{eq}}$, represent the equilibrium concentration differences at the two interfaces. According to the CA simulations, the concentration gradients at the two interfaces, $\partial_{x}C_{\gamma }{|}_{{\gamma \delta }^{+}}$ and $\partial_{x}C_{\gamma }{|}_{{\gamma L}^{-}}$, increase with decreasing temperature. On the other hand, based on the Fe-C phase diagram, the equilibrium concentration difference $C_{\gamma ,\delta /\gamma }^{\mathrm{eq}}-C_{\delta ,\delta /\gamma }^{\mathrm{eq}}$ at the δ/γ interface decreases, while $C_{L,L/\gamma }^{\mathrm{eq}}-C_{\gamma ,L/\gamma }^{\mathrm{eq}}$ at the L/γ interface increases with decreasing holding temperature. As a result, the ratio of $\partial_{x}C_{\gamma }{|}_{{\gamma \delta }^{+}}/(C_{\gamma ,\delta /\gamma }^{\mathrm{eq}}-C_{\delta ,\delta /\gamma }^{\mathrm{eq}})$ at the δ/γ interface increases remarkably, while $\partial_{x}C_{\gamma }{|}_{{\gamma L}^{-}}/(C_{L,L/\gamma }^{\mathrm{eq}}-C_{\gamma ,L/\gamma }^{\mathrm{eq}})$ at the L/γ interface keeps almost unchanged as the holding temperature decreases.

Figure 2a also shows that *a*_δγ_ > *a*_Lγ_ is valid for all holding temperatures when Ω_δ_ = Ω_L_ = 0. When a non-zero supersaturation of Ω_δ_ = Ω_L_ = 0.25 is applied, however, there exists a transition temperature *T*_tr_ as shown in Figure 2b. For *T*_0_ < *T*_tr_, the *a*_δγ_ profile is higher than that of *a*_Lγ_. In contrast, when *T*_0_ > *T*_tr_, the opposite situation, i.e., *a*_δγ_ < *a*_Lγ_, is observed. According to the simulation results in Figure 1, the supersaturation has a more significant influence on the growth rate at the L/γ interface than that at the δ/γ interface. Therefore, comparing with the case of Ω = 0 in Figure 2a, the *a*_Lγ_ profile in Figure 2b is distinctly higher, while the *a*_δγ_ curve only rises slightly. Accordingly, a transition temperature *T*_tr_ must exist, at which the condition *a*_δγ_ = *a*_Lγ_ holds. Conversely, for a certain holding temperature there exists a transition supersaturation, Ω_tr_.

Simulations were performed to investigate the relationship between transition supersaturation and holding temperature. For a given temperature, the γ-phase growth was simulated at different supersaturations. If the width of the γ-phase growing into the liquid phase, *d*_Lγ_, equals that in the δ-phase, *d*_δγ_, the transition supersaturation, Ω_tr_, is reached. Figure 3 displays the simulated Ω_tr_ varying with the holding temperature. Results of the analytical model [11] are also included for comparison. As shown, Ω_tr_ increases non-linearly with decreasing temperature. For a given holding temperature and Ω > Ω_tr_, *a*_Lγ_ > *a*_δγ_ holds. The results in Figure 3 also show a good agreement between CA simulation and analytical prediction.

**Figure 4:**
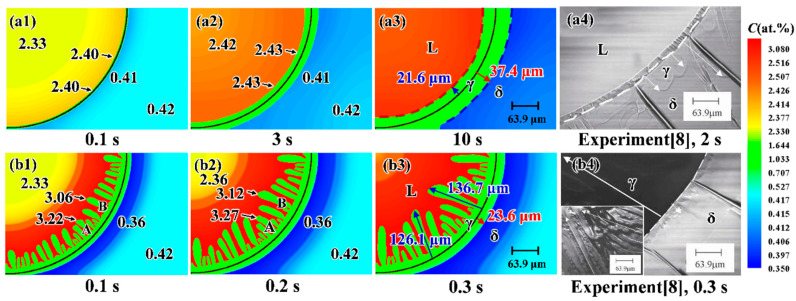
Simulated and experimental [8] microstructures with cooling rates of (**a1**–**a4**) 10 K/min and (**b1**–**b4**) 100 K/min. The black line in (**a1**–**a3**,**b1**–**b3**) and the white dashed line in (**a4**,**b4**) indicate the positions of the initial γ nucleus. The black numbers in (**a1**–**a2**,**b1**–**b2**) represent local actual concentrations. “A” and “B” in (**b1**,**b2**) denote different cellular arms of the γ-phase. The blue and red numbers in (**a3**,**b3**) represent the width of the γ-phase grown into the L- and δ-phases, respectively.

Phelan et al. [8] performed an in situ observation experiment using HTLSCM for studying the peritectic growth kinetics transition at different cooling rates. Simulations are run using the conditions of Phelan et al.’s experiment [8]. The computation domain consists of a 450 × 300 mesh. The initial microstructure includes a δ-phase rim and an inner liquid pool. The γ-nuclei with an initial thickness of 2 μm are assigned between the L- and δ-phases. The initial concentrations of the three phases were set to the equilibrium concentrations at *T*_p_. The local temperature decreases with a radial temperature gradient of 20 K/mm. Figure 4 presents the microstructural evolution during peritectic transformation with cooling rates of 10 K/min (Figure 4(a1–a4)) and 100 K/min (Figure 4(b1–b4)). The black line indicates the initial γ-phase position. Figure 4(a4,b4) present the experimental microstructures at 10 K/min after 2 s and 100 K/min after 0.3 s, respectively [8]. In Figure 4(b4), the inlay micrograph presents the cellular γ-phase morphology that grows into the L-phase. As seen in Figure 4, the local actual liquid concentration ahead of the L/γ interface increases with increasing transformation time. In Figure 4(a1,a2) with a lower cooling rate, the L/γ interface propagates with a planar morphology and the local actual liquid concentrations at different locations ahead of the L/γ interface are nearly identical. When the cooling rate is increased to 100 K/min, however, the L/γ interface evolves from a planar to a cellular morphology and the concentration distribution ahead of the L/γ interface becomes inhomogeneous. As seen in Figure 4(b1,b2), the local actual liquid concentration ahead of arm “B” is smaller than that ahead of arm “A”. According to Equation (2), the lower local liquid concentration, $C_{L,L/\gamma }^{*}$, will yield a higher driving force for the γ-phase growth. Therefore, the tip of arm “B” propagates faster than arm “A”. Moreover, the growth of arm “B” rejects solute atoms into the L-phase and the solute atoms will diffuse to the vicinity of arm “A”, which further suppresses the growth of arm “A”. Finally, the lengths of cellular γ-phase arms become more and more inhomogeneous.

For the cooling rate of 10 K/min, after growing for 1 s, the simulated L/γ interface velocity is ~7.1 μm/s, which is close to the experimental measurement (~7.8 μm/s). After 0.3 s at 100 K/min, the γ-phase thickness grown into the δ-phase is ~23.6 μm obtained by simulation (Figure 4(b3)), and ~26.3 μm as measured experimentally (Figure 4(b4)), respectively. It can be seen that *d*_δγ_ is larger than *d*_Lγ_ at 10 K/min, Figure 4(a3), while the inverse relationship is observed at 100 K/min, Figure 4(b3). It is understandable that the interface moving velocity is determined by the ratio of the diffusion flux and the concentration difference, $-D_{i}\partial_{x}C_{i}/(C_{j,j/\gamma }^{\mathrm{eq}}-C_{\gamma ,j/\gamma }^{\mathrm{eq}})\left(i=\gamma ,\delta ,L;j=\delta ,L\right)$. In the case of 10 K/min at 10 s, the estimated diffusion flux across the L/γ interface (~1.6 at.%μm/s) is slightly larger than the diffusion flux across the δ/γ interface (~0.8 at.%μm/s); the concentration differences at the L/γ and δ/γ interfaces are 1.7 at.% and 0.4 at.%, respectively. As a result, the L/γ interface migrates (~1.0 μm/s) slower than the δ/γ interface (~2.0 μm/s). At a higher cooling rate (100 K/min), the diffusion flux across the L/γ interface increases remarkably to ~640 at.%μm/s, while it only increases to ~16 at.%μm/s at the δ/γ interface. The concentration differences at the L/γ and δ/γ interfaces are 2.2 at.% and 0.3 at.%, respectively. Therefore, the L/γ interface migration velocity (~291 μm/s) becomes higher than the δ/γ interface (~53 μm/s). The simulated morphologies and growth kinetics at the two cooling rates agree reasonably with the experimental observations [8].

### 3.2. Microstructural Evolution during Peritectic Reaction

**Figure 5:**
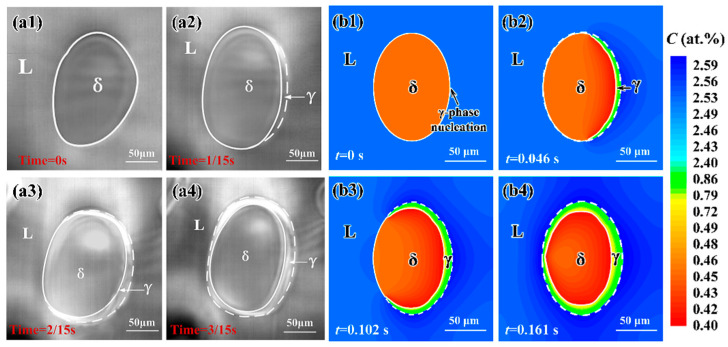
(**a1**–**a4**): Experimentally observed [17] and (**b1**–**b4**): simulated microstructural evolution during peritectic solidification of an Fe-0.83 at.%C alloy with a cooling rate of 0.1 K/s. The white solid and dashed lines indicate the positions of the δ/γ and L/γ interfaces, respectively.

Peritectic reaction is a more complex process as compared to the peritectic transformation due to the existence of the triple junction. CA simulations are carried out to study the microstructural evolution of an Fe-C alloy during peritectic reaction under a continuous cooling condition. The computation domain consists of a 1000 × 1000 mesh with a mesh size of Δ*x* = 0.2 μm. Based on the experimental condition [17], the temperature in the simulation domain is set to be uniform at *T*_0_ = 1765 K and the system is cooled down with a cooling rate of 0.1 K/s. The initial microstructure includes the L-phase and an elliptic δ-phase in the center. The γ-phase nucleates at the L/δ interface on the right-hand side. Figure 5 presents the experimentally observed [17] and simulated microstructural evolution during peritectic solidification of an Fe-0.83 at.%C alloy. The white solid and dashed lines in Figure 5 mark out the positions of the δ/γ and L/γ interfaces, respectively. As shown in Figure 5, the γ-phase propagates along the L/δ interface through the peritectic reaction (L + δ→γ). In the CA simulation at *t* = 0.161 s (Figure 5(b4)), the γ-phase completely encircles the δ-phase, resulting in the δ-phase isolation from the L-phase. The subsequent γ-phase growth into the δ- and L-phases proceeds through the peritectic transformation as discussed in Section 3.1 above.

The mean migration velocity of the L/γ/δ triple junction can be calculated by the ratio of migration distance to the peritectic reaction time. The simulated mean propagation velocity of the triple junction along the L/δ interface is ~1.1 mm/s, which is close to the experimentally measured velocity (~1.36 mm/s). Moreover, during the peritectic reaction period, the intervenient γ-phase grows in thickness through peritectic transformation. As seen in Figure 5(a4,b4), the γ-phase on the right-hand side is obviously thicker than that on the left-hand side, which is due to the fact that the peritectic transformation time on the right-hand side is longer than that on the left-hand side. The simulated microstructural evolution and γ-phase growth kinetics during peritectic solidification agree well with the experimental observations [17].

## 4. Conclusions

A multi-phase CA model previously proposed by the present authors is extended and applied for the quantitative simulation of peritectic transition, including both peritectic transformation and peritectic reaction in Fe-C alloys. The CA simulations of peritectic transformation show that the supersaturation accelerates the γ-phase growth remarkably at the L/γ interface. However, the effect of supersaturation on the δ/γ interface moving velocity is unconspicuous. At the δ/γ interface, the parabolic rate constant, *a*_δγ_, increases significantly as the holding temperature decreases, while at the L/γ interface, *a*_Lγ_ stays almost unchanged. In the case of zero supersaturation, *a*_δγ_ is always larger than *a*_Lγ_ when the holding temperatures are below the peritectic temperature. In the case of non-zero supersaturations, there exists a transition temperature, *T*_tr_. For the holding temperatures of *T*_0_ < *T*_tr_, the γ-phase growth kinetics into the δ-phase is faster than that into liquid, while at the holding temperatures of *T*_0_ > *T*_tr_, the opposite behavior is observed. For a given temperature, there exists a transition supersaturation, and it increases non-linearly with decreasing temperature. Microstructural evolution during peritectic transformation has been simulated with different cooling rates. At low cooling rates, the δ/γ interface moves faster than L/γ interface. At high cooling rates, the γ-phase grows more into the L-phase with a cellular morphology. Microstructural evolution during peritectic reaction has been simulated with an experimental cooling condition. It is observed that the γ-phase propagates along the L/δ interface and finally encircles the δ-phase. Meanwhile, the intervenient γ-phase grows in thickness through peritectic transformation. The CA simulations are in good agreement with the results obtained from the analytical model and experimental data.

## Data Availability

All data contained within the article.
